# 140. Variations in Rates of Antimicrobial Use in Primary Care, in Relation to the Presence of Chronic Diseases, Québec, Canada, 2014-2017

**DOI:** 10.1093/ofid/ofab466.342

**Published:** 2021-12-04

**Authors:** Elise Fortin, Caroline Sirois, Caroline Quach, Marc Simard, Sonia Jean, Alejandra Irace-Cima, Nadine Magali-Ufitinema

**Affiliations:** 1 Institut national de santé public du Quebec, Quebec, Quebec, Canada; 2 Université Laval, Quebec, Quebec, Canada; 3 Montreal University, Montreal, Quebec, Canada; 4 Institut national de santé publique du Québec, Quebec, Quebec, Canada; 5 Ministère de la Santé et des Services sociaux, Montreal, Quebec, Canada

## Abstract

**Background:**

Chronic diseases may increase one’s risk of infection and ensuing complications, which in turn may lower clinicians’ tolerance threshold for antimicrobial prescription, while potential drug interactions may limit therapeutic options. Objective of the study was to measure the impact of chronic diseases on the rates of antimicrobial use in the Province of Québec.

**Methods:**

Individuals covered by the public drug insurance plan between April 2014 and March 2017 were included in our cohort to describe rates of antimicrobial dispensing per 1,000 person-years, per age group (0-17 years old, 18-64 years old and 65 years old or over) and category of chronic disease (respiratory, cardiovascular, diabetes, mental disorder, none of these). For 2014-2017, ratios of extended-to-narrow spectrum antimicrobials were computed and multivariate Poisson regression was used to measure the impact of categories of chronic diseases on rates of total antimicrobial dispensing (in prescriptions and defined daily doses).

**Results:**

A total of 1 259 833 children-years, 5 281 026 person-years between 18 and 64 years and 3 841 359 person-years 65 years or older were included in the study. Ratios of extended-to-narrow spectrum antimicrobials varied from 3.1 (adults 18-64 years old with no chronic disease) to 14.6 (children with no chronic disease); ratios for individuals with chronic diseases were lower in children but higher in adults. Adults with chronic respiratory diseases were twice more exposed to antimicrobials (increase of 109%) than those with none of the studied diseases (62% increase in children). In adults, antimicrobial use was also 48% higher in presence of a mental disorder (22% in children), 40% higher with diabetes (102% in children) and 31% higher with a cardiovascular disease (no data in children). These differences were all statistically significant (α = 0,05).

**Conclusion:**

In Québec, antimicrobial dispensation was more frequent for individuals with at least one chronic disease. This raises the question of how much antimicrobial use can be reduced or improved to limit the selection of resistant bacteria.

**Disclosures:**

**All Authors**: No reported disclosures

